# Development and Validation of a Weigh-in-Motion Methodology for Railway Tracks

**DOI:** 10.3390/s22051976

**Published:** 2022-03-03

**Authors:** Bruno Pintão, Araliya Mosleh, Cecilia Vale, Pedro Montenegro, Pedro Costa

**Affiliations:** CONSTRUCT—LESE, Faculty of Engineering, University of Porto, 4200-465 Porto, Portugal; amosleh@fe.up.pt (A.M.); cvale@fe.up.pt (C.V.); paires@fe.up.pt (P.M.); pmbcosta@reit.up.pt (P.C.)

**Keywords:** weigh-in-motion system, estimation of static load, railway vehicle, strain gauges, wayside condition monitoring

## Abstract

In railways, weigh-in-motion (WIM) systems are composed of a series of sensors designed to capture and record the dynamic vertical forces applied by the passing train over the rail. From these forces, with specific algorithms, it is possible to estimate axle weights, wagon weights, the total train weight, vehicle speed, etc. Infrastructure managers have a particular interest in identifying these parameters for comparing real weights with permissible limits to warn when the train is overloaded. WIM is also particularly important for controlling non-uniform axle loads since it may damage the infrastructure and increase the risk of derailment. Hence, the real-time assessment of the axle loads of railway vehicles is of great interest for the protection of railways, planning track maintenance actions and for safety during the train operation. Although weigh-in-motion systems are used for the purpose of assessing the static loads enforced by the train onto the infrastructure, the present study proposes a new approach to deal with the issue. In this paper, a WIM algorithm developed for ballasted tracks is proposed and validated with synthetic data from trains that run in the Portuguese railway network. The proposed methodology to estimate the wheel static load is successfully accomplished, as the load falls within the confidence interval. This study constitutes a step forward in the development of WIM systems capable of estimating the weight of the train in motion. From the results, the algorithm is validated, demonstrating its potential for real-world application.

## 1. Introduction

Train WIM is of great interest in controlling axles and vehicle loads, detecting wheel defects and predicting train derailment in order to guarantee acceptable train operations and safety [1,2,3,4,5]. The presence of high loads in the track induces damage in the track components, such as rails, sleepers and ballast, which has implications in maintenance periodicity [6]. WIM systems are essential for freight wagons with overloaded or unbalanced loads, as it leads to damages to the infrastructure on the one hand and enhances the risk of derailment on the other hand. In recent decades, the development of efficient WIM systems with track data acquisition has been one of the important topics that has attracted the attention of the railway industry and scientific researchers.

In general, there are two approaches to estimate the dynamic train loads: (i) by direct measurement at the contact between wheel and rail [7]; and (ii) by indirect measurement on the rail of the forces applied by the vehicle to the infrastructure [8].

In the first approach, sensors are installed directly on the wheels, axle or bogies of the rail vehicle for measuring the dynamic loads. These types of devices are defined as onboard monitoring systems. Bernal et al. [9] reviewed recent onboard condition monitoring sensors, systems, methods and techniques, aiming to define the present state of the art and its potential application for freight wagons. By using this approach, Kanehara and Fujioka [10] estimate vehicle axle loads using electrical strain gauges. Uhl [11] proposes a force identification method based on the transfer function between the dynamic responses measured on specific vehicle components, such as the acceleration at the bogies and the vertical contact forces between wheel and rail [12]. Despite recent developments on onboard systems, there may still be some technical limitations related to the power supply, communication system and adaptability of the systems to different types of trains [13]. Moreover, onboard detection methods are also commonly used to monitor the track condition, and not exclusively for WIM estimation. Therefore, in contrast to onboard devices, wayside measurement systems are currently an ideal solution to estimate the static load of the wagon when the train is in motion, since results can be obtained for each train, avoiding the installation of devices in every vehicle.

The second approach uses measurements from wayside monitoring systems, which assess the train loads from the dynamic track response. This indirect method can be categorised by: (1) WIM on the track; (2) bridge weigh-in-motion, (B-WIM) [14,15,16,17]. In this approach, loads from multiple vehicles can be estimated by using a single given section of the rail track or bridge. For higher speeds, Allotta et al. [18] developed a methodology to determine the wheel and the axle loads of the vehicles. Their algorithm was capable of estimating the vehicle load by considering the measured vertical forces on the sleepers.

Usually, WIM systems are based on rail strain measurements that use electrical strain gauges installed on the rails to obtain signals that are proportional to the applied loads. Strain gauges are configured to amplify very low measured strains (increase the signal-to-noise ratio) and are usually installed in the rail’s neutral axis. In addition, shear forces and the bending moments are easily inferred by strain gauges (SGs), as they are direct consequences of the load applied in the track. Onat et al. approaches an interesting model based on the interpretation of wheel angular and vehicle translational velocities instead of measurements from the track or instrumented wheelsets [19]. Although most applications report the use of electrical strain gauges [20], including some recent work on the design of optimal strain gauges [21], some emerging technologies using piezoelectric sensors [22,23,24] and fiber optic sensors [25] are also available in the literature.

Previous research [3,26] states that the load assessed by dynamic WIM systems is composed of two components: the quasi-static component, which corresponds to the distribution of the weight on the different wheelsets, and the dynamic component, which results from the dynamic effects induced by the train. When it comes to a perfect track, the quasi-static component is preeminent, since the loads applied by the train wheelset are approximately constant over time and independent of the vehicle’s speed given the smooth condition of the platform. However, in the real situation, even if the track is in good condition, due to the discrete support of the rails, the evaluated load on the track is a dynamic load. Considering all of these complex effects, it is necessary to propose a system to estimate the static load for each wheelset when the train is moving.

Despite a large number of publications on railway conditional monitoring [27,28,29], to the knowledge of the authors, reported literature on automatic WIM has been limited so far. The novelty of this paper is to propose and validate a WIM methodology to estimate the wheel and axle static loads when the train is in motion. The dynamic weighing methodology was tested in several scenarios in order to evaluate the influence of the railpad stiffness and damping, WIM installation position and contact stiffness on the dynamic load. Finally, the methodology was tested, with synthetic values reachable by dynamic wayside weighing systems composed by strain gauges.

It should be highlighted that the preliminary research on this topic was developed by Mosleh et al. [26], who demonstrated the influence of the speed and unevenness profile quality on the estimated static load. The validation of the proposed WIM methodology herein, considering different types of trains, is a clear step forward in terms of the effectiveness of the proposed methodology, which allowed for a complete implementation for real-world application.

## 2. Layout Scheme of the WIM System

**Figure 1:**
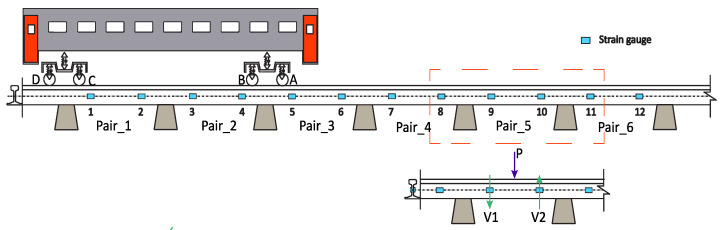
Schematic of the WIM system composed of six pairs of strain gauges.

To obtain the wheel static load, it is necessary to establish a monitoring system that can measure the dynamic component of the vehicle–track interaction forces to estimate the weight of the wheel under moving conditions. In this paper, a WIM methodology is proposed and validated using two types of trains running at different speeds and under different track conditions. In Figure 1, a wayside WIM system consisting of six pairs of strain gauges installed along the rail is presented. The strain gauges are placed along a total length of 3.6 m, which considers seven sleepers at equal distances of 0.6 m.

The proposed methodology uses the measurements from the strain gauges of the WIM system. The dynamic axle load (*P*) from a passage of a train can be evaluated by the difference between the shear (*V*_1_ and *V*_2_) (see Figure 1) from two consecutive sections, as indicated in Equation (1).

(1) $$V_{1}-V_{2}=P$$

## 3. Description of the Train–Track Coupling Model

A dynamic analysis of the train–track systems is a complex task because it comprises the interconnection between two sub-systems, the track and the train, that interact with each other through the wheel–rail contact interface [30,31]. To reduce the computational time, several authors have implemented integral transformation methods to obtain dynamic system responses [32,33,34,35,36]. In this approach, the track is simulated as an infinitive and invariant system placed on the ground, modelled as a Winkler foundation. The dynamic of the track is solved in the frequency–wavenumber domain and then inverted for the space–time domain. The train–track interaction problem is solved in the frequency domain, considering that both sub-systems are perfectly coupled through compatibility equations [37].

### 3.1. Track Model

**Figure 2:**
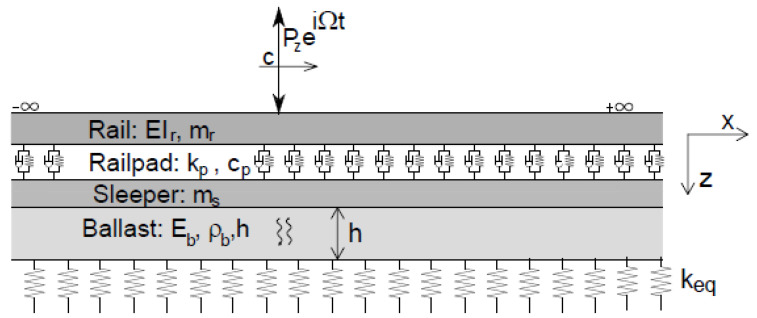
Track model.

The numerical model of the track, presented in Figure 2, is implemented in MATLAB^®^ [38] software, and consists of a three-layer model, which includes the ballast, the sleeper and the rail. The rail is simulated by an Euler–Bernoulli beam uniformly supported by a spring–dashpot system representing the rail pads. Sleepers are modelled as a distributed mass layer, while the ballast is modelled considering a one-direction wave propagation layer. In this research, a 2.5D FEM-BEM model previously developed and validated by Alves Costa et al. is used [29]. The ground is defined as a Winkler model. The mechanical properties of the track are presented in Table 1. More details regarding the track modelling, including railpad damping, ballast hysteretic damping and the equivalent stiffness of the foundation, can be found in the study by Mosleh et al. [26].

### 3.2. Train Model

**Figure 3:**
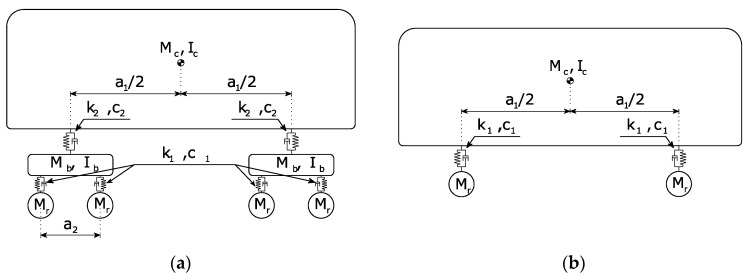
Dynamic models of the railway vehicle: (**a**) Alfa Pendular; (**b**) freight wagon.

Two types of vehicles are adopted in this study: the Alfa Pendular train and a freight wagon, as demonstrated in Figure 3. The models are based on the work by Zhai and Cai [39]; the geometrical and mechanical properties used are presented in Table 2, attained from [40]. 

### 3.3. Train–Track Interaction

To model the train–rail interaction, it is necessary to fulfil the requirements of displacement compatibility and the equilibrium conditions at the contact point between the rolling stock and the track. In the study, the compatibility requirement denotes calculating Equation (2) in different time instants *t*:

(2) $$u_{v,i}=u_{r}\left(x=vt+s_{i}\right)+\Delta u\left(t+\frac{s_{i}}{v}\right)+\frac{P_{i}\left(t\right)}{K_{h}}$$

where $u_{v,i}$ represents the vertical displacements of the contact point, $u_{r}$ represents the vertical displacement of the rail at contact point *i*, Δ*u* is the unevenness profile of the rail, $s_{i}$ is the location of the contact point *i* at *t* = 0 s and *v* is the vehicle speed. In the present approach, the track irregularity is considered as a dynamic excitation source, represented by *P*(*t*). The last term in Equation (2) considers the Hertzian deformation at the wheel–rail contact, where $K_{h}$ is the linearised contact stiffness and $P_{i}$ is the dynamic interaction load developed at contact point *i*. A linearised stiffness is used to avoid solving a nonlinear problem. The linearised contact stiffness can be calculated by Equation (3) according to [41]:

(3) $$K_{h}=\sqrt[{3}]{\frac{3r_{eq}QE^{2}}{2{\left(1-\nu^{2}\right)}^{2}}}$$

where $r_{eq}$ is the equivalent radius of curvature, *Q* is the static load on each wheel of the train, *E* is the Young’s modulus of elasticity (200 GPa) and ν is the Poisson’s coefficient. The value attained in Equation (3) should be multiplied by two when considering full modelling. Since no significant variations in the dynamic wheel–rail contact load are expected to occur for the scenarios analysed in this work, a linearised stiffness approximation may be acceptable [31]. However, for other scenarios, such as the wheel flats, the nonlinear contact between the wheel and the rail should be adopted [6].

### 3.4. Unevenness Profile

The dynamic load transmitted by the moving vehicle into the track highly depends on the unevenness track profile. In this research study, only the vertical irregularities of the rail are considered because the aim of the algorithm is to evaluate vertical loads. The generation method used for the unevenness profile is provided by Mosleh et al. [26], and consists of a stationary stochastic process described by a spectral density function (PSD), defined by the Federal Railway Administration (FRA) [42,43], that can be written as:

(4) $$S_{rzz}\left(k1\right)=\frac{10^{-7}Ak_{3}^{2}\left(k_{1}^{2}+k_{2}^{2}\right)}{k_{1}^{4}\left(k_{1}^{2}+k_{3}^{2}\right)}\;\;\;\;\;k_{1}=\frac{2\pi }{\lambda }$$

where $k_{2}$ and $k_{3}$ are constants values given as 0.1464 rad/m and 0.8168 rad/m, respectively, $k_{1}$ is the wavenumber dependent on the cyclic spatial frequency of irregularity, λ is the wavelength randomly generated between 1 m and 30 m and A is a parameter, given in Table 3, that represents the track quality.

**Figure 4:**
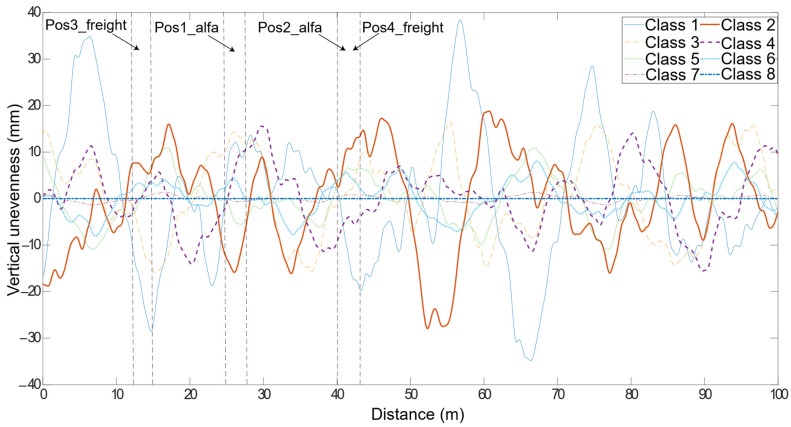
Unevenness profiles of the rail, including different classes proposed by FRA.

By considering Equation (4) and the values in Table 3, track profiles with a 100 m length have been generated, and are shown in Figure 4 for different track classes. In this figure, pos1_Alfa, pos2_Alfa, pos3_freight and pos4_freight are the possible positions of the WIM system installation. In the following section, the influence of the position of the installed WIM system is also analysed by considering two possible different locations for the installation of the wayside monitoring system.

### 3.5. Methodology for the Dynamic Load Assessment

As previously mentioned, the proposed algorithm to calculate the static axle loads is based on the assessment of two shear measurements from consecutive sections. In the wavenumber–frequency domain, shear (*V*) can be obtained from the third derivative of the rail displacement, described by Equation (5):

(5) $$V\left(k_{1},\omega \right)=EIik_{1}^{3}d_{r}^{G}\left(k_{1},\omega \right)\times L\left(k_{1},\omega \right)$$

where $L\left(k_{1},\omega \right)$ represents the loading function, and is acquired from the solution of the train track interaction, *EI* is the rail bending stiffness, “*i*” is the counter that varies between 1 and the number of wavelengths that were implemented in the modeling of the unevenness profile and $d_{r}^{G}\left(k_{1},\omega \right)$ is the vertical displacement of the rail for unitary loading. By neglecting the inertial forces generated on the rail, and by applying an inverse Fourier transform over the space *P*(*t*), the dynamic axle load is given by

(6) $$P\left(t\right)=\sum_{i=1}^{n}\left[\begin{array}{c} \frac{1}{2\pi }\int_{-\infty }^{+\infty }EIik_{1}^{3}d_{r}^{G}\left(k_{1},\omega =\Omega_{i}-k_{i}v\right)\times L_{i}\left(k_{1},\Omega_{i}\right)e^{-i\left(s_{2}-s_{3}\right)k_{1}}dk_{1}\\ -\frac{1}{\pi \left(s_{2}-s_{3}\right)}\int_{-\infty }^{+\infty }k_{p}^{*}\left[d_{r}^{G}\left(k_{1},\omega =\Omega_{i}-k_{i}v\right)-d_{s}^{G}\left(k_{1},\omega =\Omega_{i}-k_{i}v\right)\right]\times L_{i}\left(k_{i},\Omega_{i}\right)\frac{\sin\left(k_{1}\frac{s_{2}-s_{3}}{2}\right)}{k_{1}}e^{-i\left(s_{2}-s_{3}\right)k_{1}}dk_{1} \end{array}\right]e^{i\Omega_{i}t}$$

where $L_{i}$ is the dynamic interaction load for each wavelength, and Ω and *v* are the frequency and speed.

## 4. Sensitivity Analysis Regarding the Axle Dynamic Loads

This section shows the influence of different vehicle speeds, different unevenness profiles of the track and the Hertzian linearised wheel–rail contact spring stiffness on the evaluated axle dynamic load. Moreover, several instances of the stiffness and damping of the rail pads are used to evaluate the dynamic load of the wheelset. In the study, two types of vehicles that run in the Portuguese railway network are considered: the Alfa Pendular passenger train and a freight wagon. To simplify the numerical analyses, only one vehicle car per train is considered. The axle static loads are 130 kN and 214 kN for Alfa Pendular (four axles per vehicle) and the freight wagon (two axles per vehicle), respectively. Train speeds of 5 m/s (18 km/h), 10 m/s (36 km/h), 20 m/s (72 km/h) and 40 m/s (144 km/h) are considered in this study, while the track profiles are generated considering the six track classes proposed by the FRA and described in Table 3. Additionally, two other classes are considered to be taken into account: a very good track (class 7) with *A* = 1.02 $m^{3}$/rad, and a smooth track (class 8) with *A* = 0.

To evaluate the influence of the Hertzian contact stiffness $(K_{h}$), three values of $K_{h}$ are considered for each train: $K_{h,est}$ representing the contact stiffness calculated by Equation (3) with the vehicle axle static load; $K_{h,din+}$ and $K_{h,din-}$ representing the contact stiffness associated, respectively, with the maximum and the minimum dynamic load attained for the speed of 40 m/s, as is the case when the dynamic load has a greater variation. By considering the properties of the wheel, the value of $K_{h,est}$ is 1.186 × 10^6^ kN/m for the Alfa Pendular and 1.401 × 10^6^ kN/m for the freight wagon, and the Hertzian contact stiffnesses $K_{h,din+}$ and $K_{h,din-}$ calculated for each track quality class are indicated in Table 4.

To evaluate the influence of the stiffness and damping of the rail pads, three values of rail pads stiffness are considered, $k_{p}$: 40, 100 and 500 kN/mm, as well as two values of damping, $c_{p}$: 10 and 20 kN.s/m. The correlation table was attained in Excel. From the results obtained in the 188 calculations, the analysis of the correlation table (Table 5) allows for an evaluation of the relationship between the train speed (*V*), track class (*C*), the type of train (*T*), the contact stiffness (*C_S_*), the railpad stiffness (*k_p_*) and railpad damping (*c_p_*) in the calculated dynamic load (*DL*).

From this table, it is possible to observe that the dynamic load has a strong correlation with the type of train, which means that the dynamic properties of the train significantly influence the predicted dynamic load. The dynamic load also has a strong correlation, but with a negative value with the contact stiffness, which means that the dynamic load increases with a reduction in the contact stiffness.

**Figure 5:**
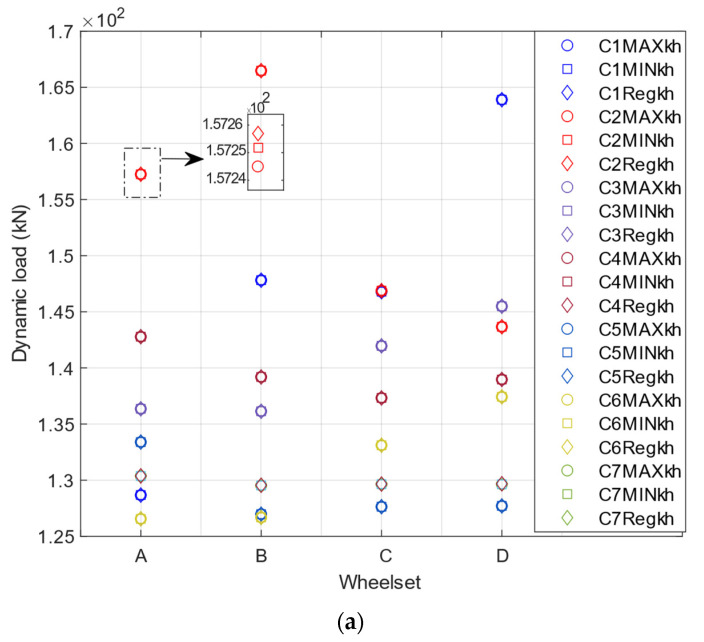

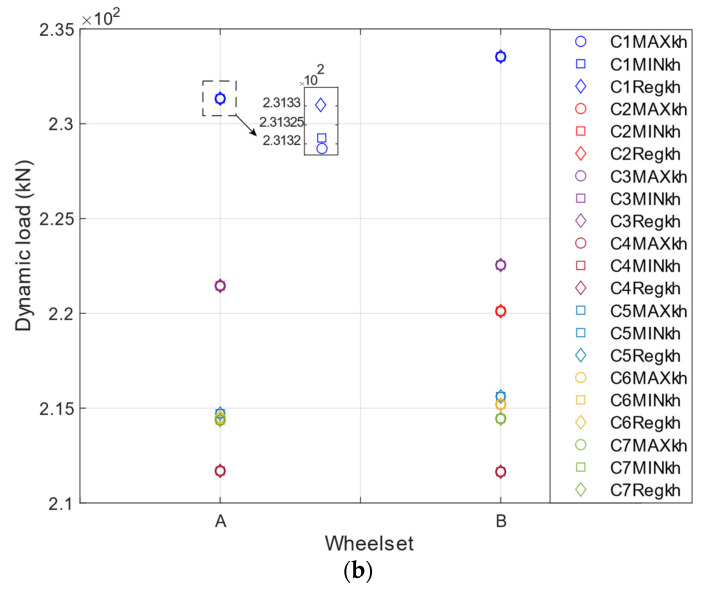
Dynamic load for different values of contact stiffness: (**a**) Alfa Pendular; (**b**) freight wagon.

Figure 5 shows the influence of different contact stiffnesses on the evaluated dynamic load, where the load is depicted for speed = 40 m/s. By looking in detail at the dynamic load, the percentage difference between the highest and the lowest dynamic load (Figure 5) is limited to 0.03% for all track quality classes, which is an insignificant value. For the vehicle speeds and track profiles in this analysis, this small difference shows that contact linearisation in the track–vehicle interaction modelling is acceptable. Note that the evaluated dynamic load is the mean value of the loads obtained from six pairs of strain gauges. In Figure 5, “C” stands for the “class” of the track, “Maxkh” is the value of $K_{h}$ for the maximum dynamic load on the class evaluated and “Minkh” is the value of $K_{h}$ for the minimum dynamic load on the class evaluated.

**Figure 6:**
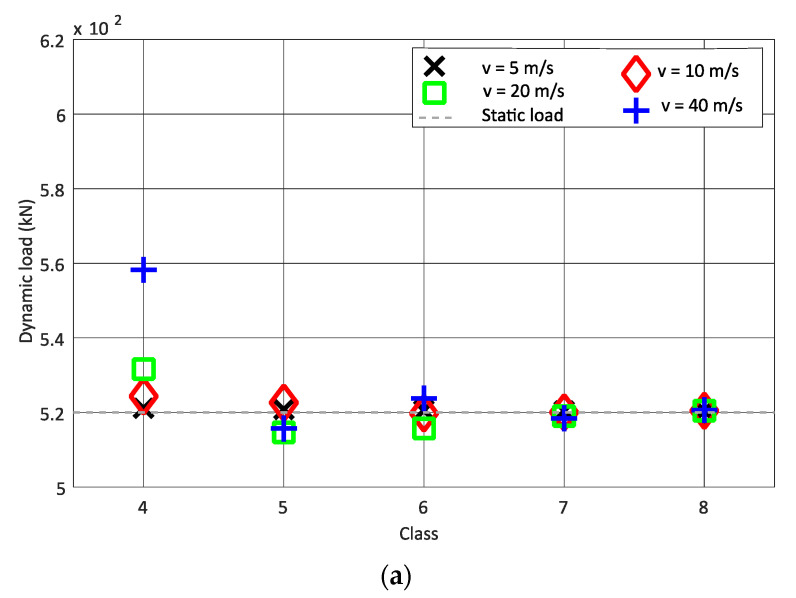

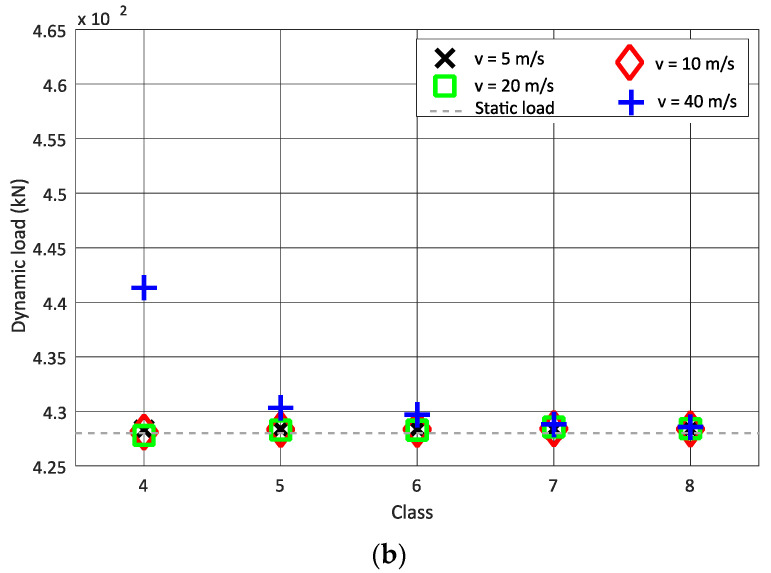
Influence of vehicle speed on the dynamic load: (**a**) Alfa Pendular; (**b**) freight train.

Although the correlation between the vehicle speed and dynamic load is positive, it is not as strong as the correlation between the dynamic load and type of train. In Figure 6, the dynamic load for each vehicle speed and track class from class 4 to class 8 is shown for a better understanding of the Alfa Pendular vehicle and freight wagon. From the results presented in Figure 6, it is possible to conclude that, for the same speed, as the track quality improves (the class type increases from 4 to 8), the mean obtained dynamic load value is closer to the static load. In this scenario, for each class, one synthetic profile is generated and the dynamic load is obtained from six pairs of strain gauges. Therefore, the mean value is calculated from the six loads obtained for each speed. The influence of speed is only shown for classes 4 to 8, since classes 4 to 8 have better track quality compared to classes 1 to 3; therefore, the maximum permitted speed is higher in these classes.

**Figure 7:**
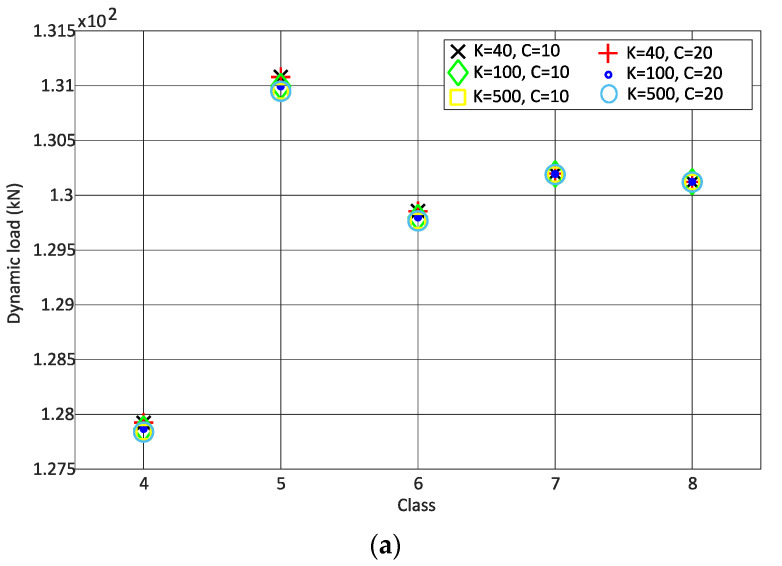

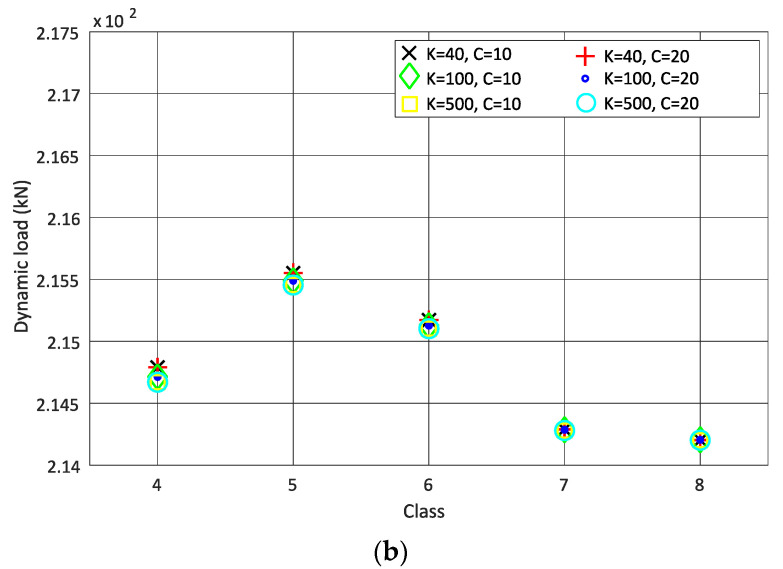
Influence of the rail pads stiffness and damping on dynamic load: (**a**) Alfa Pendular; (**b**) freight wagon.

Figure 7 shows the dynamic load versus the track classes due to different stiffness and damping coefficients of rail pads due for the passage of the Alfa Pendular vehicle and the freight wagon, respectively, with a speed of 20 m/s. These figures show that the variation of stiffness and damping for rail pads does not have a significant influence on the calculated dynamic load, as shown in the correlation table. For the same reasons as stated for Figure 6, only classes 4 to 8 are taken into account.

**Figure 8:**
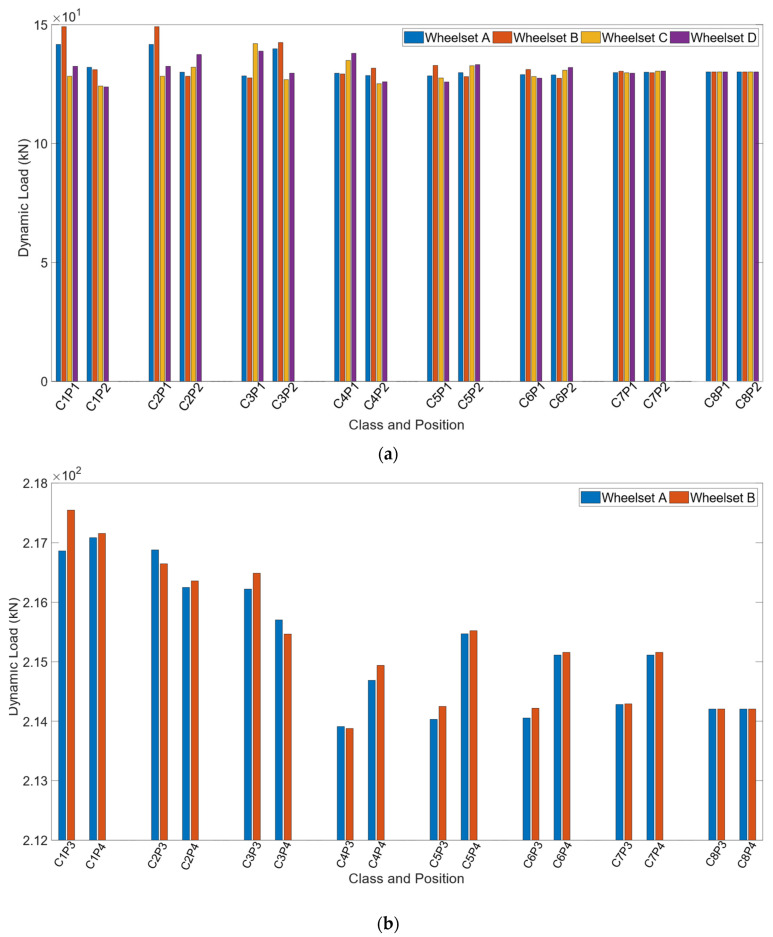
Influence of the position of the system installation in the assessment of the dynamic load for (**a**) Alfa Pendular; (**b**) freight wagon.

In this study, the influence of the position of the installed WIM system is also analysed by considering two possible different locations for the installation of the wayside monitoring system. For the Alfa Pendular vehicle, the two considered sections are: 25–28 m (pos1_Alfa) and 40–43 m (pos2_Alfa), whereas, for the freight train, the sections are 12–15 m (pos3_freight) and 40–43 m (pos4_freight). These positions are presented in Figure 4. The numerical results are shown in Figure 8. In the x axle, “C” refers to the track quality class and “P” the position of the monitoring device, i.e., “C1 P2” means class 1 in position 2. From these figures, it can be inferred that the dynamic load may be sensitive to the position of the monitoring system in tracks. As an example, when the vehicle passes through the location 25–28 m (pos1_Alfa), the mean value of the axle dynamic load (wheel B, as presented in Figure 1) obtained from six pairs of strain gauges for the Alfa Pendular vehicle that corresponds to class 1 is 149 kN, whereas the same load for wheel B when the vehicle passes through position 40–43 m (pos2_Alfa) is 130 kN. These differences in the obtained dynamic load may be due to the fact that the unevenness profile on the track is different and that the evaluated dynamic load from one position is different from the dynamic load obtained at the other position. Therefore, the location of the installed WIM system may influence the estimated static load.

## 5. Evaluation of Static Loads through a WIM System

### 5.1. Methodology

**Figure 9:**
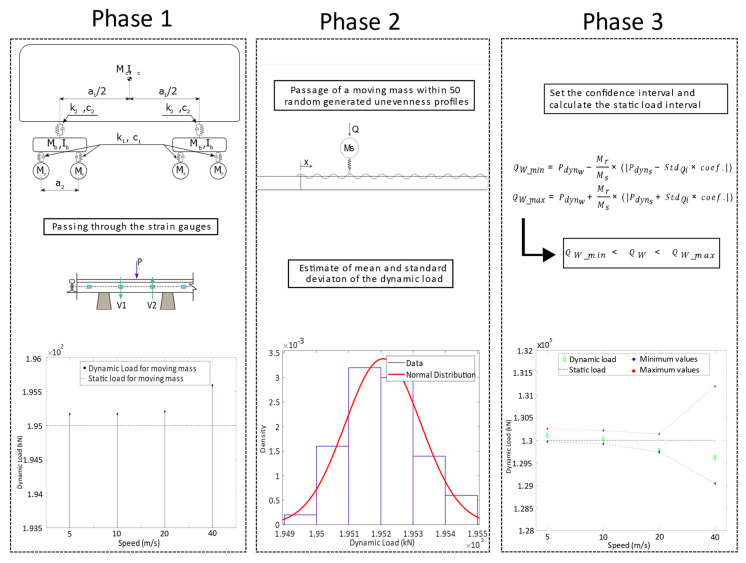
Schematic representation of the proposed WIM methodology.

By the results presented in the previous section, it can be concluded that a reasonable estimation of the static load can be obtained by a large number of measurements from strain gauge pairs installed in the track. However, for optimising the cost of the monitoring devices and for facilitating the installation and maintenance activities, sensors in the track should be the minimum requirement. Thus, it should be possible to estimate the vehicle static load by considering limited pairs of strain gauges. As a consequence of the reduction in the number of sensors in the track, the static loads cannot be calculated by a deterministic framework, but a statistical procedure needs to be considered to define an interval of confidence. The methodology is schematically indicated in Figure 9.

As can be seen, the proposed statistical procedure considers three phases, described below.

1st Phase—A train passes over a monitoring system consisting of a limited number of pairs of strain gauges installed in the track that measure the shear (*V*) on each position of the gauges. Once the shear force in two consecutive positions is obtained, the dynamic load (*P*) is calculated by the difference between the two values, as indicated in Figure 1. In other words, when the train is passing over the WIM system, the average value of loads (from six pairs of strain gauges) of each wheelset ($P_{dyn_{w}}$) is assessed by Equation (1). The unsprung masses of each wheelset of the vehicle ($M_{r}$) are identified as well. $M_{r}$ can be obtained from a database collecting the properties of the rolling stock. The train speed is computed from experimental data from the passage of the train;

2nd Phase—By considering the passage of moving mass over a large enough number of track unevenness profiles, the normal distribution of the dynamic load values is identified: the mean value and standard deviation of the distributed dynamic loads. Moreover, by subtracting the static load of an arbitrary wheelset (Q) to the mean value of the distribution of dynamic loads ($Q_{i\text{\_}mean}$), the effect of dynamic load ($P_{dyn_{s}}$) is calculated. In the presented study, 50 dynamic calculations were considered. It should be highlighted that the unevenness profiles in this step should be compatible with the geometrical quality class of the track and for the speed assessed in the first phase;

3rd Phase—In the last phase, the wheel static load ($Q_{W}$), is calculated from the load measured by the WIM system ($P_{dyn_{w}}$). The static load is estimated by removing the dynamic component of the load. This step is performed by subtracting the $P_{dyn_{w}}$ from the dynamic load component evaluated for the passage of a known wheelset ($P_{dyn_{s}}$) evaluated in phase 2, which is affected by a correction factor that corresponds to the ratio between the wagon wheelset mass ($M_{r}$) and the mass of the known wheelset ($M_{s}$) as indicated in Equation (7), to calculate the minimum and the maximum static load, which are, respectively, $Q_{W\text{\_}min}$ and $Q_{W\text{\_}max}$.

(7) $$\begin{array}{c} Q_{W\text{\_}min}=P_{dyn_{w}}-\frac{M_{r}}{M_{s}}\times \left(\left|P_{dyn_{s}}-Std_{Qi}\times coef.\right|\right)\\ Q_{W\text{\_}max}=P_{dyn_{w}}+\frac{M_{r}}{M_{s}}\times \left(\left|P_{dyn_{s}}+Std_{Qi}\times coef.\right|\right) \end{array}$$

The interval of confidence of the WIM is considered to be 95% for reliable results, and the coefficient is 1.96. Moreover, $M_{r}$ (the wheelset mass) is considered to be 1711 kg for the Alfa Pendular and 1246.52 kg for the freight train.

### 5.2. Numerical Study

In this numerical study, the described methodology is applied to estimate the static load from the passage of two trains over a WIM system. The analysed trains are the same as the study in the sensitivity analysis described in Section 5. The track unevenness profile is artificially generated by considering the characteristics of class 7. The wheelset static load is considered as 130 kN and 214 kN for Alfa Pendular vehicle and freight wagon.

**Figure 10:**
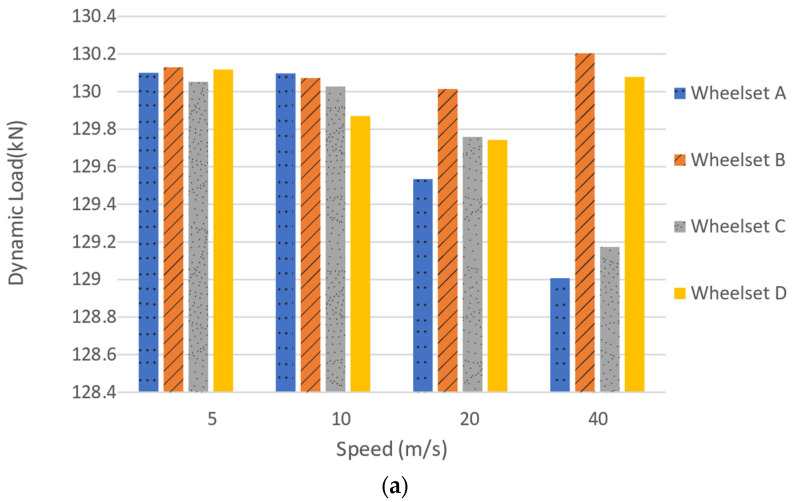

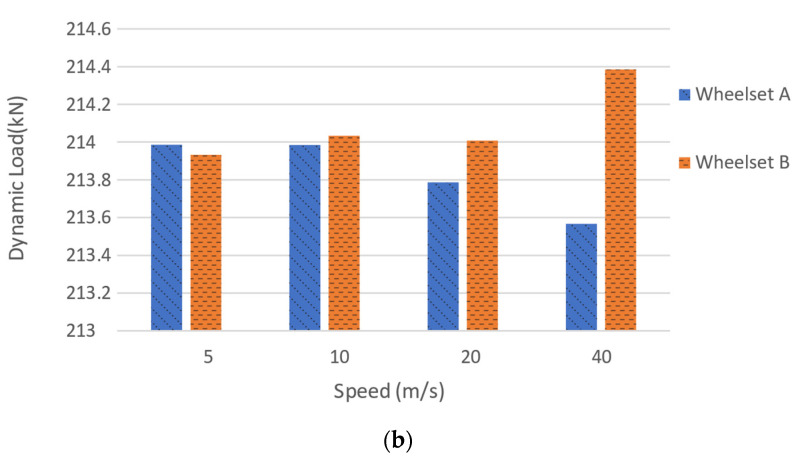
Wheelset dynamic load considering different speeds for (**a**) the Alfa Pendular and (**b**) freight wagon.

1st Phase—After applying the first step of the calculation procedure shown in Figure 9, the dynamic vehicle load ($P_{dyn_{w}}$) is obtained for each speed. Figure 10 shows the wheelset dynamic load considering different speeds for the Alfa Pendular and freight wagon.

Figure 10 shows that, even by considering a track with good geometry conditions (class 7), increasing the speed has an influence on the evaluated dynamic load. In the case where the track is perfectly smooth, the loads acquired by the dynamic weighing system are very similar to the static loads transmitted by the wheelsets to the rail (Figure 8). However, this is no longer accurate for the real track, where the perfectly smooth surface is not achieved. The load imposed by the wheelset on the track is dependent on the unevenness profile of the track, which gives rise to an increase in dynamic loads over quasi-static loads (as shown in Figure 10). Due to this increase in interaction loads, the track degradation process increases gradually with the number of rail vehicle passages over the track;

2nd Phase—The second phase of the methodology shown in Figure 9, requires the generation of the statistic distribution of the dynamic load of moving mass, with a significant number of unevenness profiles. In this study, 50 profiles of class 7 are generated, and the statistical distribution is generated for the speeds of 5, 10, 20 and 40 m/s. 

**Figure 11:**
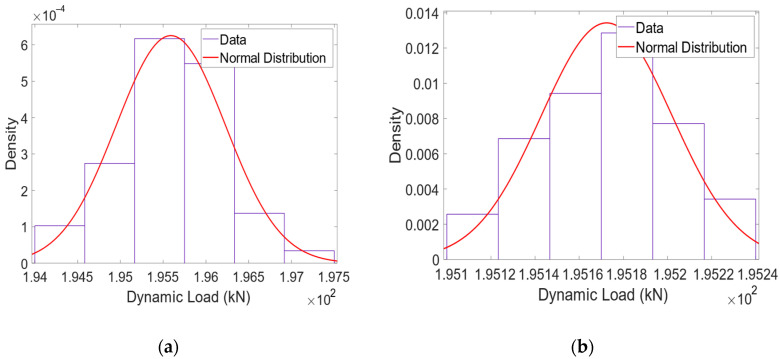

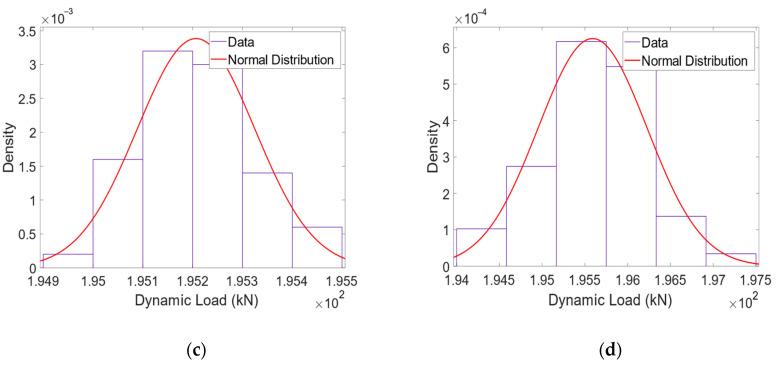
Normal distribution of dynamic loads for different speeds: (**a**) 5 m/s; (**b**) 10 m/s; (**c**) 20 m/s; (**d**) 40 m/s.

Considering the mass of the wheelset $M_{s}$, and the quasi-static load Q, the average dynamic load of the 50 passages of the moving mass for the different speeds is plotted. Figure 11 shows the dynamic load distribution of the 50 passages of the moving mass on the class 7 track for the different speeds. Then, the mean value ($Q_{i-mean}$) and standard deviation $(Std_{Qi}$) of the dynamic load of a single moving mass system, considering 50 unevenness profiles, is calculated. 

The dynamic load effect for one single wheelset ($P_{dyn_{s}}$) passing through 50 unevenness profiles is calculated by the following equation:

(8) $$P_{dyn_{s}}=Q_{i-mean}-Q$$

where $Q_{i-mean}$ is the mean value for distributed loads for 50 dynamic loads for the passage of one single wheelset;

3rd Phase—In the last step, the interval of the static load for each vehicle wheelset is estimated. The calculation of the confidence interval for the passage of the moving mass system at a speed of 40 m/s with the normal distribution function is presented in Figure 11d. For the normal distribution for the passage of moving mass at 40 m/s, the mean value ($Q_{i-mean}$) is 195,592 N and the standard deviation ($Std_{Qi}$) is calculated as 644.15. It should be noted that the quasi-static load (Q) imposed by the moving mass system is 195 kN, which means that there is a difference of $P_{dyn_{s}}$ = 592 N between the average dynamic load value and the static load value of the moving mass. When the vehicle of the Alfa Pendular is running at a speed of 40 m/s along the track, the measured dynamic load ($P_{dyn_{w}}$) for axle A is 129,006 kN. The static load for axle A ($Q_{W}$) is calculated from the measured dynamic load for the Alfa Pendular axle ($P_{dyn_{w}}$ = 129,006 kN), subtracted from the effect of dynamic load for one axle ($P_{dyn_{s}}$ = 592 N), considering the mass of the Alfa Pendular axle ($M_{r}$ = 1711 kg) and the moving mass ($M_{s}$ = 2003 kg).

**Figure 12:**
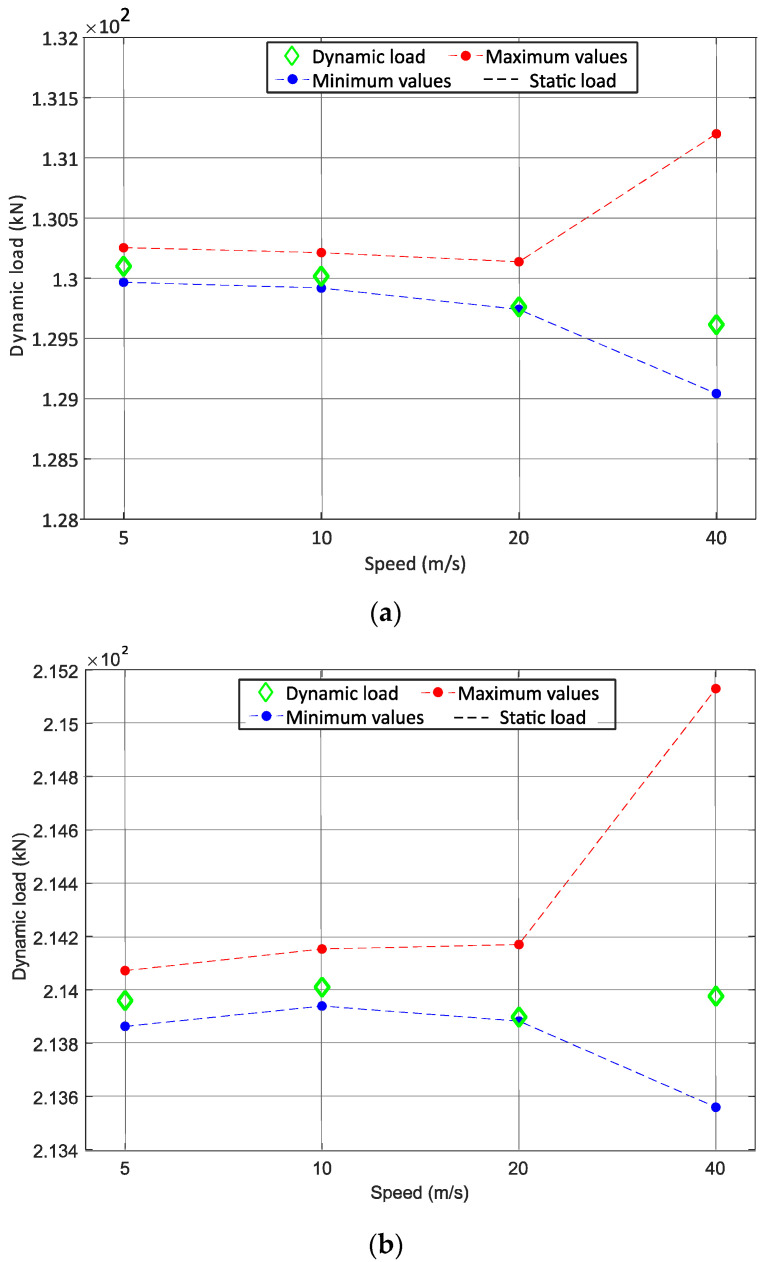
Graphical representation of the confidence interval (**a**): Alfa Pendular vehicle, (**b**): freight wagon.

The upper and lower limits of the static load range for the Alfa Pendular and the freight train are calculated, as shown in Equation (7), and with the results in Figure 12, respectively. The values of the static load transmitted by each wheelset of the vehicle to the track ($Q_{w}$) must fall within the confidence interval $Q_{W\text{\_}min}$ and $Q_{W\text{\_}max}$, which is calculated in Equation (7).

Note that the lines of the minimum and maximum value of the confidence interval are only suggestive, since the study is carried out only for the speeds of 5 m/s, 10 m/s, 20 m/s and 40 m/s. As expected, the methodology proposed to estimate the static load analysis is successfully accomplished as the dynamic load falls within the confidence interval.

## 6. Validation of the Proposed Methodology

**Figure 13:**
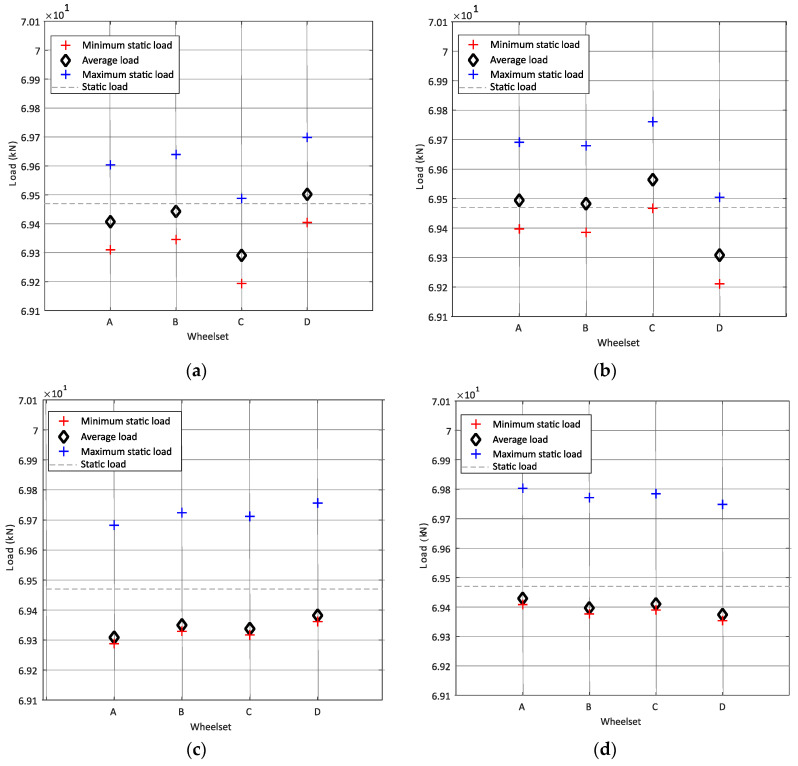
Interval of static loads for (**a**): speed = 10 m/s considering unevenness profile 1, (**b**): speed = 10 m/s considering unevenness profile 2, (**c**): speed = 20 m/s considering unevenness profile 1, (**d**): speed = 20 m/s considering unevenness profile 2.

To validate the proposed methodology, synthetic data from the passage of a wagon (presented in Figure 1) are generated. More details regarding synthetic data generation can be found in the following references [40,44] and represent the usual condition of a Portuguese railway line of class 7. The wheel static load is set as 69.470 kN. The methodology is considered to define the interval of the static load for the speed of 10 m/s and 20 m/s, and two unevenness profiles corresponding to class 7 are considered for each passage. Figure 13 shows that, for the speed of 10 m/s and 20 m/s, the average load per each wheel is contained in the interval of confidence calculated by Equation (7).

From the results presented in this section, it is possible to conclude that the proposed methodology in this research study can be used for wayside monitoring systems to estimate the weight of the train in motion.

## 7. Conclusions

The proposal considered in this article is a three-phase methodology to evaluate train static loads in WIM from a wayside monitoring system. After describing the numerical train–track models, a parametric study is presented to evaluate the influence of the speed, irregularity profile, contact stiffness and railpads stiffness and damping on the dynamic load imposed by the trains on the track. Based on the results, the following conclusions can be drawn:

- The dynamic load has a strong correlation with the type of train, which means that the dynamic properties of the train significantly influence the predicted dynamic load;
- The dynamic load also has a strong correlation with the contact stiffness; however, the contact linearisation in the track–vehicle interaction modelling is acceptable for the vehicle speeds and track profiles in the analysis;
- For the same speed, as the track quality improves (the class type increases from 4 to 8), the mean dynamic load value obtained from six positions of strain gauges is closer to the static load;
- The variation of stiffness and damping for rail pads does not have a significant influence of the calculation of the dynamic load;
- The dynamic load may be sensitive to the position of the monitoring system in tracks. These differences in the obtained dynamic load may be due to the fact that the unevenness profile of the track is different and the evaluated dynamic load from one position is different to the dynamic load obtained at the other position.

A reasonable estimation of the static load can be obtained when a significantly large number of strain gauge pairs are installed in the track. For optimising the cost of the monitoring devices and for facilitating the installation and maintenance activities, sensors in the track should be the minimum requirement. Consequently, when calculating the static load from the WIM system with fewer sensors, a statistical correlation must be considered, which means that the estimation of the interval of the static load should consider a certain level of confidence for each one of the vehicle wheelsets.

The methodology was applied and validated with synthetic data from an Alfa Pendular train moving on a Portuguese railway track, demonstrating the potential of the WIM methodology for real-world applications. Moreover, for the final development of the proposed approach, experimental validation is necessary.

## Figures and Tables

**Table 1:** Mechanical properties of the track model [26].

Rail (UIC-60)					
$A\;(m^{2}$)	$\rho \;(\mathrm{kg}/m^{3}$)	$I\;(m^{4}$)	ν (−)	$E\;(\mathrm{kN}/m^{2}$)	
0.01534	7850	6.11 × 10^−5^	0.3	2 × 10^8^	
Railpads		Sleepers	Ballast	Foundation	
$c_{p}$ (kN s/m)	$k_{p}$ (kN/m)	ρ (kg/m)	$\rho \;(\mathrm{kg}/m^{3}$)	G (MPa)	$\rho \;(\mathrm{kg}/m^{3}$)
30	2 × 10^5^	525	1550	20	1900

**Table 2:** Geometric and mechanical properties of the trains [40].

Properties		Alfa Pendular Vehicle	Freight Wagon
Box	Mass—*M_c_* (kg)	35,640	41,100
	Pitch moment of inertia—*I_c_* (kg·m^2^)	1,475,000	673,322.46
Secondary suspension	Stiffness—*k*_2_ (kN/m)	734,832	0
	Damping—*c*_2_ (kN·s/m)	35	0
Bogie	Mass—*M_b_* (kg)	2829	16,739
	Pitch moment of inertia—*I_b_* (kg·m^2^)	1931.49	0
Primary suspension	Stiffness—*k*_1_ (kN/m)	1,652,820	1,860,000
	Damping—*c*_1_ (Ns/m)	16,739	16,739
Axle	Mass—*M_r_* (kg)	1711	1246.52
	Static load per axle—*Q* (kN)	130	214
Dimensions	Longitudinal distance between wheelsets—a_2_ (m)	2.7	-
	Longitudinal distance between bogies—a_1_ (m)	19	6

**Table 3:** Values of A proposed by FRA.

Class	1	2	3	4	5	6
$A\;(m^{3}/\mathrm{rad}$)	660.079	376.229	208.841	116.856	65.929	37.505

**Table 4:** Contact stiffness for maximum and minimum dynamic loads.

Alfa Pendular			Freight Train		
Class	Kh	kN/m	Class	Kh	kN/m
**Class 1**	$K_{h,din+}$	1.282 × 10^6^	**Class 1**	$K_{h,din+}$	1.442 × 10^6^
	$K_{h,din-}$	1.182 × 10^6^		$K_{h,din-}$	1.438 × 10^6^
**Class 2**	$K_{h,din+}$	1.288 × 10^6^	**Class 2**	$K_{h,din+}$	1.417 × 10^6^
	$K_{h,din-}$	1.227 × 10^6^		$K_{h,din-}$	1.414 × 10^6^
**Class 3**	$K_{h,din+}$	1.232 × 10^6^	**Class 3**	$K_{h,din+}$	1.419 × 10^6^
	$K_{h,din-}$	1.205 × 10^6^		$K_{h,din-}$	1.417 × 10^6^
**Class 4**	$K_{h,din+}$	1.224 × 10^6^	**Class 4**	$K_{h,din+}$	1.396 × 10^6^
	$K_{h,din-}$	1.208 × 10^6^		$K_{h,din-}$	1.396 × 10^6^
**Class 5**	$K_{h,din+}$	1.197 × 10^6^	**Class 5**	$K_{h,din+}$	1.404 × 10^6^
	$K_{h,din-}$	1.177 × 10^6^		$K_{h,din-}$	1.402 × 10^6^
**Class 6**	$K_{h,din+}$	1.209 × 10^6^	**Class 6**	$K_{h,din+}$	1.403 × 10^6^
	$K_{h,din-}$	1.176 × 10^6^		$K_{h,din-}$	1.402 × 10^6^
**Class 7**	$K_{h,din+}$	1.187 × 10^6^	**Class 7**	$K_{h,din+}$	1.402 × 10^6^
	$K_{h,din-}$	1.185 × 10^6^		$K_{h,din-}$	1.402 × 10^6^

**Table 5:** Correlation matrix between train–track properties.

	*V*	*C*	*T*	*C_S_*	*k_p_*	*c_p_*	*DL*
*V*	1						
*C*	−5.37 × 10^−2^	1					
*T*	−9.87 × 10^−17^	−3.34 × 10^−17^	1				
*C_S_*	4.15 × 10^−2^	−2.48 × 10^−2^	9.99 × 10^−2^	1			
*k_p_*	−1.11 × 10^−2^	−1.53 × 10^−3^	−1.65 × 10^−17^	−1.11 × 10^−3^	1		
*c_p_*	−2.19 × 10^−1^	−3.04 × 10^−2^	2.50 × 10^−17^	2.34 × 10^−2^	−4.11 × 10^−2^	1	
*DL*	1.45 × 10^−1^	−1.18 × 10^−1^	9.46 × 10^−1^	−9.17 × 10^−1^	−5.05 × 10^−3^	7.99 × 10^−2^	1

## Data Availability

Not applicable.

## References

[1] Mayer R., Poulikakos L., Lees A.R., Heutschi K., Kalivoda M., Soltic P. (2012). Reducing the environmental impact of road and rail vehicles. Environ. Impact Assess. Rev..

[2] Molodova M., Li Z., Núñez A., Dollevoet R. (2014). Automatic Detection of Squats in Railway Infrastructure. IEEE Trans. Intell. Transp. Syst..

[3] Mosleh A., Costa P., Calçada R. (2020). Development of a Low-Cost Trackside System for Weighing in Motion and Wheel Defects Detection. Int. J. Railw. Res..

[4] Chen S., Feng D., Sun Z. (2021). Reliability-based vehicle weight limit determination for urban bridge network subjected to stochastic traffic flow considering vehicle-bridge coupling. Eng. Struct..

[5] Xu L., Zhai W. (2020). Train–track coupled dynamics analysis: System spatial variation on geometry, physics and mechanics. Railw. Eng. Sci..

[6] Vale C. (2021). Wheel Flats in the Dynamic Behavior of Ballasted and Slab Railway Tracks. Appl. Sci..

[7] Bosso N., Gugliotta A., Zampieri N. (2018). Wheel flat detection algorithm for onboard diagnostic. Measurement.

[8] Mosleh A., Meixedo A., Costa P., Calçada R. Trackside Monitoring Solution for Weighing in Motion of Rolling Stock. Proceedings of the TESTE2019—2nd Conference on Testing and Experimentations in Civil Engineering—Proceedings.

[9] Bernal E., Spiryagin M., Cole C. (2019). Onboard Condition Monitoring Sensors, Systems and Techniques for Freight Railway Vehicles: A Review. IEEE Sens. J..

[10] Kanehara H., Fujioka T. (2002). Measuring rail/wheel contact points of running railway vehicles. Wear.

[11] Uhl T. (2007). The inverse identification problem and its technical application. Arch. Appl. Mech..

[12] Neto J., Montenegro P.A., Vale C., Calçada R. (2020). Evaluation of the train running safety under crosswinds—A numerical study on the influence of the wind speed and orientation considering the normative Chinese Hat Model. Int. J. Rail Transp..

[13] Vale C., Bonifácio C., Seabra J., Calçada R., Mazzino N., Elisa M., Terribile S., Anguita D., Fumeo E., Saborido C. (2016). Novel Efficient Technologies in Europe for Axle Bearing Condition Monitoring—The MAXBE Project. Transp. Res. Procedia.

[14] Carraro F., Gonçalves M.S., Lopez R.H., Miguel L.F.F., Valente A.M. (2019). Weight estimation on static B-WIM algorithms: A comparative study. Eng. Struct..

[15] Pimentel R., Ribeiro D., Matos L., Mosleh A., Calçada R. (2021). Bridge Weigh-in-Motion system for the identification of train loads using fiber-optic technology. Structures.

[16] Sun Z., Siringoringo D., Fujino Y. (2021). Load-carrying capacity evaluation of girder bridge using moving vehicle. Eng. Struct..

[17] Hajializadeh D., Žnidarič A., Kalin J., OBrien E.J. (2020). Development and Testing of a Railway Bridge Weigh-in-Motion System. Appl. Sci..

[18] Allotta B., Adamio P.D., Marini L., Meli E., Pugi L., Rindi A. (2015). A New Strategy for Dynamic Weighing in Motion of Railway Vehicles. IEEE Trans. Intell. Transp. Syst..

[19] Onat A., Kayaalp B.T. (2019). A Novel Methodology for Dynamic Weigh in Motion System for Railway Vehicles With Traction. IEEE Trans. Veh. Technol..

[20] Costa B., Martins R., Santos M., Felgueiras C., Calçada R. (2017). Weighing-in-motion wireless system for sustainable railway transport. Energy Procedia.

[21] Zhou W., Abdulhakeem S., Fang C., Han T., Li G., Wu Y., Faisal Y. (2020). A new wayside method for measuring and evaluating wheel-rail contact forces and positions. Measurement.

[22] Bracciali A., Ciuffi R., Piccioli F., Knothe K. Progetto e validazione di un sensore estensimetrico multifunzionale per il binario ferroviario. In the Proceedings of XXX Congresso AIAS.

[23] Delprete C., Rosso C. (2009). An easy instrument and a methodology for the monitoring and the diagnosis of a rail. Mech. Syst. Signal Process..

[24] Sekuła K., Kołakowski P. (2012). Piezo-based weigh-in-motion system for the railway transport. Struct. Control. Health Monit..

[25] Kouroussis G., Kinet D., Moeyaert V., Dupuy J., Caucheteur C. (2016). Railway structure monitoring solutions using fibre Bragg grating sensors. Int. J. Rail Transp..

[26] Mosleh A., Costa P., Calçada R. (2020). A new strategy to estimate static loads for the dynamic weighing in motion of railway vehicles. Inst. Mech. Eng. Part F J. Rail Rapid Transit.

[27] Mohammadi M., Mosleh A., Razzaghi M., Costa P., Calçada R. (2022). Stochastic analysis of railway embankment with uncertain soil parameters using polynomial chaos expansion. Struct. Infrastruct. Eng..

[28] Costa P.M.B.A. (2011). Vibrações do Sistema via-Maciço Induzidas por Tráfego Ferroviário: Modelação Numérica e Validação Experimental.

[29] Costa P.A., Calçada R., Silva Cardoso A. (2012). Track–ground vibrations induced by railway traffic: In-situ measurements and validation of a 2.5D FEM-BEM model. Soil Dyn. Earthq. Eng..

[30] Dahlberg T. (2001). Some railroad settlement models—A critical review. Proc. Inst. Mech. Eng. Part F J. Rail Rapid Transit.

[31] Vale C., Calçada R. (2014). A dynamic vehicle-track interaction model for predicting the track degradation process. J. Infrastruct. Syst..

[32] Dieterman H.A., Metrikine A. (1996). Equivalent stiffness of a half-space interacting with a beam. Critical velocities of a moving load along the beam. Eur. J. Mech. A-Solids.

[33] Sheng X., Jones C.J.C., Thompson D. (2004). A theoretical model for ground vibration from trains generated by vertical track irregularities. J. Sound Vib..

[34] Steenbergen M.J.M.M., Metrikine A.V. (2007). The effect of the interface conditions on the dynamic response of a beam on a half-space to a moving load. Eur. J. Mech. A-Solids.

[35] Thompson D., Kouroussis G., Ntotsios E. (2019). Modelling, simulation and evaluation of ground vibration caused by rail vehicles. Veh. Syst. Dyn..

[36] Lombaert G., Degrande G., François S., Thompson D. (2015). Ground-Borne Vibration due to Railway Traffic: A Review of Excitation Mechanisms, Prediction Methods and Mitigation Measures. Notes Numer. Fluid Mech. Multidiscip. Des..

[37] Song Y., Wang Z., Liu Z., Wang R. (2021). A spatial coupling model to study dynamic performance of pantograph-catenary with vehicle-track excitation. Mech. Syst. Signal Process..

[38] Luo R. (2008). Anti-sliding control simulation of railway vehicle braking. J. Mech. Eng..

[39] Zhai W., Cai Z. (1997). Dynamic interaction between a lumped mass vehicle and a discretely supported continuous rail track. Comput. Struct..

[40] Mosleh A., Montenegro P.A., Costa P.A., Calçada R. (2021). Railway Vehicle Wheel Flat Detection with Multiple Records Using Spectral Kurtosis Analysis. Appl. Sci..

[41] Hertz H. (1882). Ueber die Berührung fester elastischer Körper [On the contact of elastic solids]. J. Für Die Reine Und Angew. Math..

[42] Fries R.H., Coffey B.M. (1990). A State-Space Approach to the Synthesis of Random Vertical and Crosslevel Rail Irregularities. J. Dyn. Syst. Meas. Control..

[43] Hamid A., Yang T.L. (1981). Analytical Description of Track Geometry Variations.

[44] Mosleh A., Montenegro P., Alves Costa P., Calçada R. (2020). An approach for wheel flat detection of railway train wheels using envelope spectrum analysis. Struct. Infrastruct. Eng..

